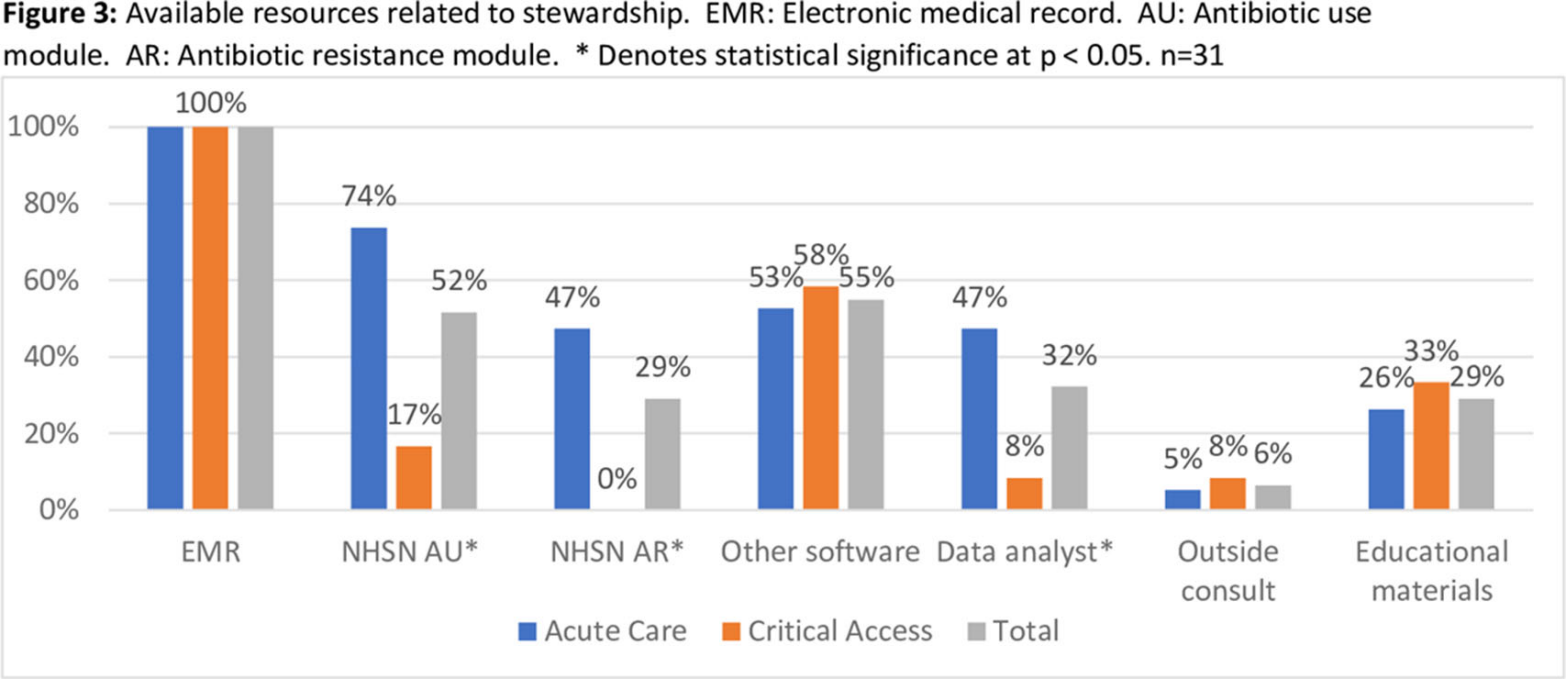# Assessing Robustness of Antimicrobial Stewardship in Colorado Hospitals and Barriers to Improvement

**DOI:** 10.1017/ash.2021.27

**Published:** 2021-07-29

**Authors:** Daniel Dodson, Matthew Kronman, Sarah Parker, Christopher Czaja

## Abstract

**Background:** Adherence to core elements of antimicrobial stewardship programs (ASPs) is increasing nationally but the robustness of programs and inclusion of pediatrics is poorly understood. We describe the details of ASP in Colorado hospitals and identify steps by which academic centers and public health departments can assist community ASPs. **Methods:** We invited ASP leaders at the 102 acute-care hospitals (ACHs) and critical-access hospitals (CAHs) in Colorado to participate in a web-based survey regarding their ASPs. Questions related to adherence to Centers for Disease Control and Prevention (CDC) core elements, barriers to improvement, desired resources, and extension to pediatrics. Enrollment began in August 2020. Hospital types were compared using the Fisher exact test. **Results:** As of January 1, 2021, 31 hospitals (30% of targeted hospitals) completed the web-based survey including 19 ACH and 12 CAH. Hospitals were distributed across the state. Median number of beds was 52 (range, 11–680). Of the responding hospitals, 87% were adherent to all CDC core elements. However, if action was defined as prospective audit and feedback or prior authorization, tracking was defined as measuring antibiotic use in days of therapy (DOT) or defined daily dose (DDD) quarterly, and reporting was defined as providing unit- or provider-specific antibiotic use reports annually. Overall adherence fell to 35% including 81% for action, 58% for tracking, and 58% for reporting. CAHs were less likely to adhere to these strict criteria than ACHs (Figure 1). In the 27 hospitals (87% of hospitals) caring for pediatric patients, adherence to a strict action for at least 1 pediatric population was 59%. Reported barriers to improved ASP were available time and personnel, information technology support, perceived concerns about provider attitudes, and education gaps (Figure 2). CAHs were less likely to use the NHSN antibiotic use or resistance modules or have a data analyst than ACHs (Figure 3). Pediatric pharmacy expertise and guidelines were often not available in hospitals caring for pediatric patients. Desired ASP resources included assistance with data analysis, access to stewardship expertise and education, and treatment guidelines, including for pediatrics. **Conclusions:** Adherence to CDC core elements of an ASP was excellent but fell dramatically when stricter criteria were used and was worse in pediatric patients. Academic centers and public health departments can assist community hospitals by providing educational resources, assistance in analyzing data including using the NHSN ED: /AR modules, and ASP expertise and clinical care guidelines including those for pediatrics.

**Funding:** No

**Disclosures:** None

**Figure 1.**
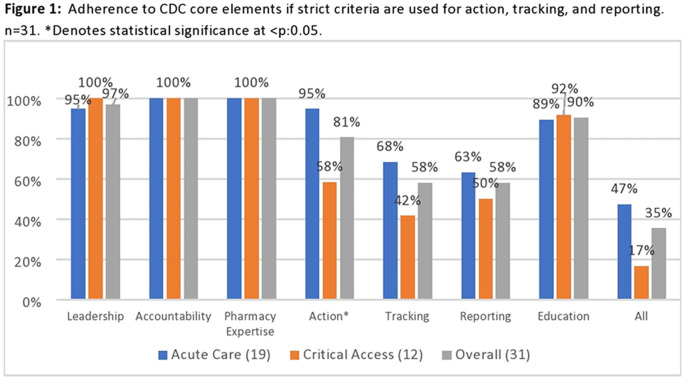

**Figure 2.**
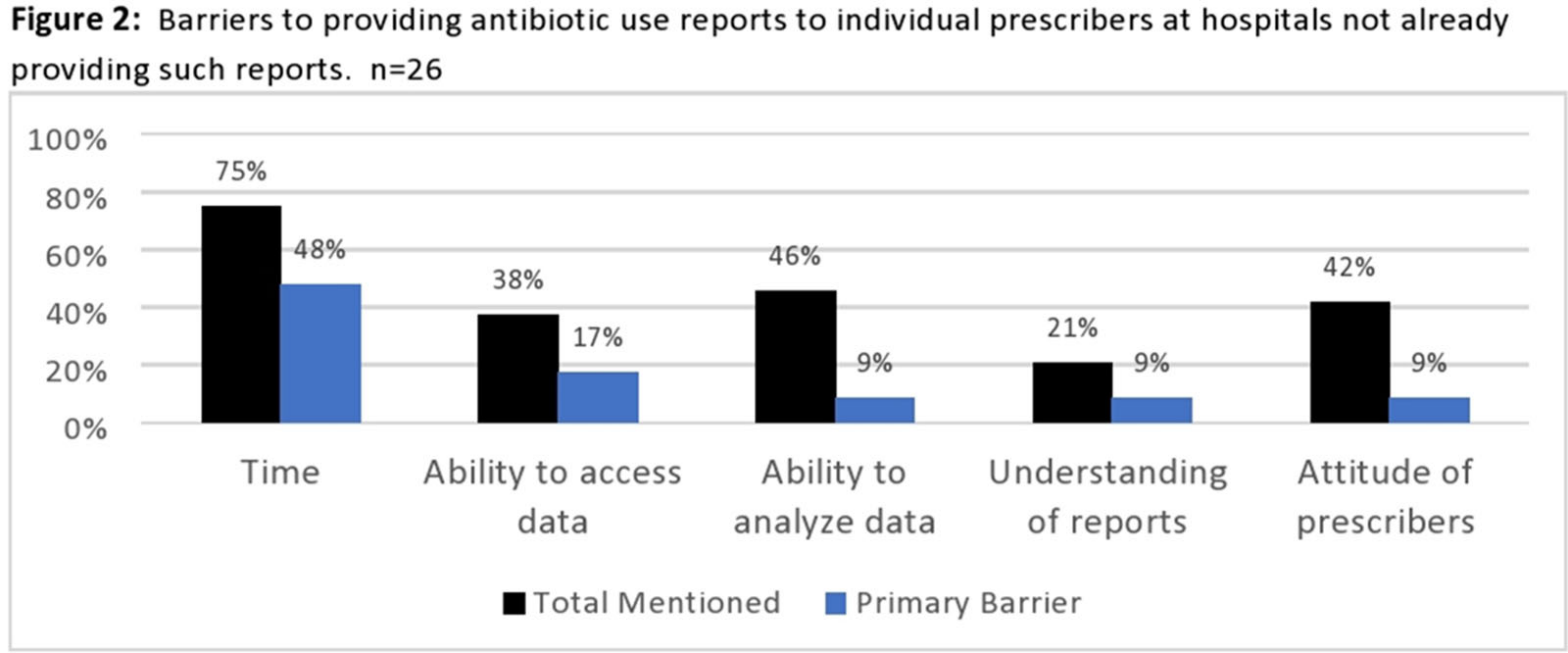

**Figure 3.**